# Validation of Hepascore as a Predictor of Liver Fibrosis in Patients with Chronic Hepatitis C Infection

**DOI:** 10.1155/2011/972759

**Published:** 2011-12-28

**Authors:** Hamid Kalantari, Hannan Hoseini, Anahita Babak, Majid Yaran

**Affiliations:** ^1^Department of Gastroenterology, Liver Diseases Research Center, Isfahan University of Medical Sciences, 81876-98191 Isfahan, Iran; ^2^Department of Gastroenterology, School of Medicine, Isfahan University of Medical Sciences, 81876-98191 Isfahan, Iran; ^3^Infectious Diseases and Tropical Medicine Research Center, Isfahan University of Medical Sciences, 81876-98191 Isfahan, Iran

## Abstract

*Introduction*. Liver biopsy is an invasive determinator for hepatic fibrosis. Serum biomarkers can probably be used as an alternative to liver biopsy in assessment of the degree of fibrosis in patients with chronic Hepatitis C. *Method*. Eighty patients with chronic Hepatitis C were included in the study using simple nonrandom sampeling metod. After fulfillment of liver biopsy, the patients were categorized according to the METAVIR Scoring system. The Hepascore algorithm is computed based on age, sex, and the serum levels of total bilirubin, *δ*-glutamyl transferase, *α*2-Macroglobulin, and hyaluronic acid. The spearman and ROC tests were used. *Results*. According to the liver biopsy results, 12, 25, 20, 7 and 16 patients had F0, F1, F2, F3, and F4, respectively. With regard to the 0.34 cut-off point Hepascore had 67%, 56%, 64%, and 56% sensitivity, specificity, respectively, positive prediction value (PPV), and negative prediction value (NPV), respectively, for diagnosis of significant fibrosis. For a Hepascore cut-off point 0.61, sensitivity, specificity, respectively, PPV and NPB 82%, 86%, 70%, and 92% in diagnosis of severe fibrosis. For a Hepascore cut-off point 0.84, sensitivity, specificity, PPV and NPB were respectively 100%, 97%, 89%, and 100% for diagnosis of cirrhosis. *Conclusion*. Hepascore has a high value in diagnosis of the level of fibrosis, particularly cirrhosis. Therefore, it can be used for primary screening of patients to determine the need for liver biopsy.

## 1. Introduction

The prevalence of hepatitis C virus (HCV) has been estimated to be 3% throughout the world. The severity of the disease is dependent on the extension of hepatic fibrosis. Liver biopsy is currently considered as the gold standard for determination of the grade of fibrosis [1]. Presence of significant fibrosis (F2–4) is accepted as the treatment indication [2, 3]. Liver biopsy is unfortunately associated with many complications for patients, including pain, bleeding, and rarely death, high costs, variations in diagnosis of histological grades, and sampling errors [4]. Furthermore, liver biopsy can provide only a view of a static or dynamic disease, and multiple biopsies are required to determine the disease progression or recurrence of fibrosis. This reveals the need for noninvasive, precise, and valid methods [5].Considerable advances have occurred in identification of nonspecific fibrosis biomarkers. The nonspecific markers include age, gender, and laboratory markers of liver damage or dysfunction (AST, ALT, *γ*-glutamyl transferase (GGT), bilirubin, hapatoglobin, platelet count, and prothrombin time), while metabolic markers are cholesterol, apoprotein A1 (Apo A1), and *α*2-macroglobulin (A2M).

So far, the index of ratio of AST to platelet count (APRI) has been the simplest test using nonspecific markers, which is valuable in predicting fibrosis [6–9].

The fibrometer test, which includes hyaluronic acid (HA), PT test, platelet count, AST, A2M, urea, and age, is to some extent efficient. Another achievement was the use of specific fibrosis biomarkers such as HA, matrix metalloproteinase 2, tissue inhibitor of matrix metalloproteinase 1, and amino-terminal peptide of type III procollagen [1, 10]. When used in combination with each other, it is known that these markers are valid in determining the liver fibrosis score [11]. Recently, some attempts have been performed to improve the nonspecific indices by use of fibrosis-specific markers. Adams et al. suggested Hepascore, which is a combination of HA, total bilirubin, GGT, A2M, age, and gender [12]. Hepascore was shown to be valid in the HCV population in Australia and then in two other groups in a European country (France) [12–14]. The aim of the current study is to compare the results of liver biopsy of patients with hepatitis C, genotype 1, in Isfahan with their Hepascore, and also to determine sensitivity, specificity, and positive and negative predictive values of Hepascore.

## 2. Materials and Methods

The participants were in the age range of 18–65, with the mean age of 35.3 ± 12.7. Out of the participants, 68 patients (85%) were males.

Among the patients with hepatitis C, genotype 1, who referred to the GI clinic or ward of Alzahra Hospital from April to October 2010 and met the inclusion criteria of the study, 80 patients were included in the study using nonrandom simple method. The inclusion criteria were the newly diagnosed patients of hepatitis C, genotype 1, who were candidates of liver biopsy. If the patients were not willing to participate in the study, they were excluded.

### 2.1. Liver Biopsy

All liver biopsies were taken under supervision of a hepatology subspecialist using an 18-gauge Menghini needle or a 16-gauge Trucut needle, with the size of ≥10 mm. The specimens were then evaluated by a single skilled pathologist for the degree of fibrosis according to the METAVIRE classification [1].

The degree of fibrosis was classified in a 0–4 scale as follows: F0: no fibrosis, F1: portal fibrosis alone, F2: portal fibrosis with rare septae, F3: portal fibrosis with many septae, and F4: cirrhosis. Grades F2, F3, and F4 indicate significant fibrosis, F3 and F4 show severe fibrosis, and F4 indicates cirrhosis.

### 2.2. Blood Samples

From all the participants, a 10 mL blood sample was obtained, and its serum was kept at −70°C. Determination of serum level of hyaluronic acid (Corgenix, USA), GGT (Biosystems, Spain), and A2M (Immundiagnostik, German) was performed using enzyme-linked protein binding assay, and the level of total bilirubin was determined using Biosystem A15 Autoanalyzer with specific reagents.

### 2.3. Calculation of Hepascore

To calculate the Hepascore, the values obtained for the four biomarkers, A2M, GGT, total bilirubin, and HA, and the age and gender of the patient are set in the following formula, which was first issued by Adam et al. [12] in 2005:

(1) $$\begin{array}{cc} Y & =\text{EXP}(-4.185818-\left(0.0249\;*\;\text{age}\right)\;\;\;\\ & \;\;\;\;+\left(0.7464\;*\;\text{sex}\right)+\left(1.0039\;*\;\text{A}2\text{M}\right)\\ & \;\;\;\;+\left(0.0302\;*\;\text{HA}\right)+\left(0.0691\;*\;\text{Bil-t}\right)\\ & \;\;\;\;\;\;-\left(0.0012*\;\text{GGT}\right)),\\ & \text{Hepascore}=\frac{Y}{\left(1+Y\right)}. \end{array}$$

The value for sex in the above formula is 1 for men and 0 for women.

### 2.4. Statistical Analysis

All data was analyzed in the SPSS software, using rho Spearman and ROC analysis. The *P* values below 0.05 were considered to be statistically significant.

## 3. Results

The range of variation, mean, and standard deviation values for HA, A2M, GGT, and bilirubin is provided in Table 1.

### 3.1. Liver Histology

The results of liver biopsy for the patients were as follows: 12 patients were F0, 25 were patients F1, 20 were patients F2, seven patients were F3, and 16 patients were F4. The frequency distribution for fibrosis severity according to the biopsy results is demonstrated in Table 2.

### 3.2. Hepascore of Patients

**Figure 1:**
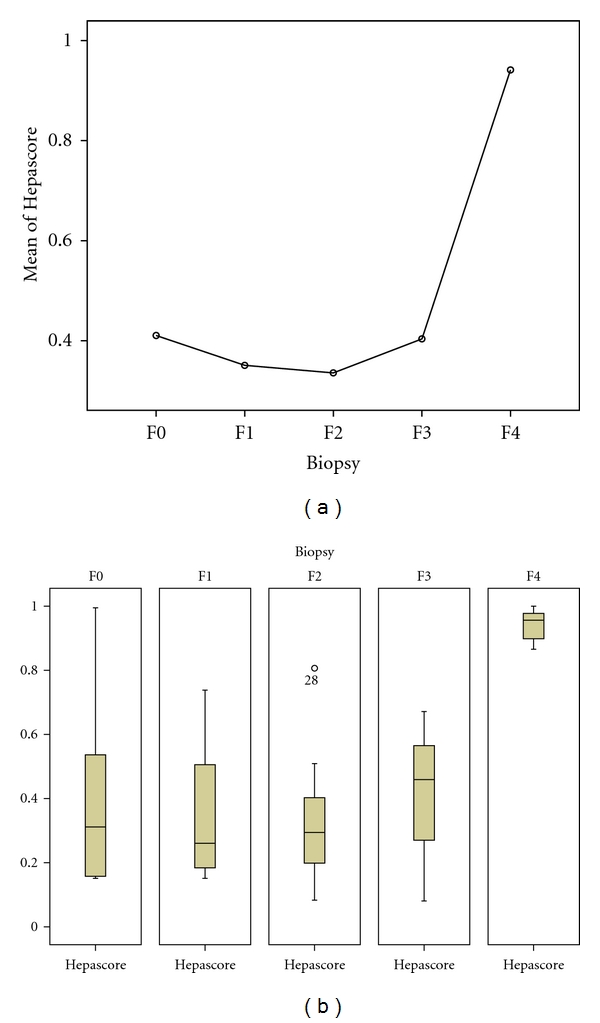
(a) Relationship between mean of Hepascore and severity of liver fibrosis. (b) Relationship between median of Hepascore and severity of liver fibrosis.

Mean and median of Hepascore in different degrees of fibrosis (according to biopsy) are shown in Figures 1(a) and 1(b), respectively.

**Correlation between Hepascore and Biopsy Results**

According to the results obtained from Spearman's correlation test, there is a relatively strong correlation between severity of fibrosis estimated by Hepascore and that determined by liver biopsy (*r* = 0.465, *P* = 0.003).

### 3.3. Sensitivity, Specificity, and Positive and Negative Predictive Values of Hepascore

**Figure 2:**
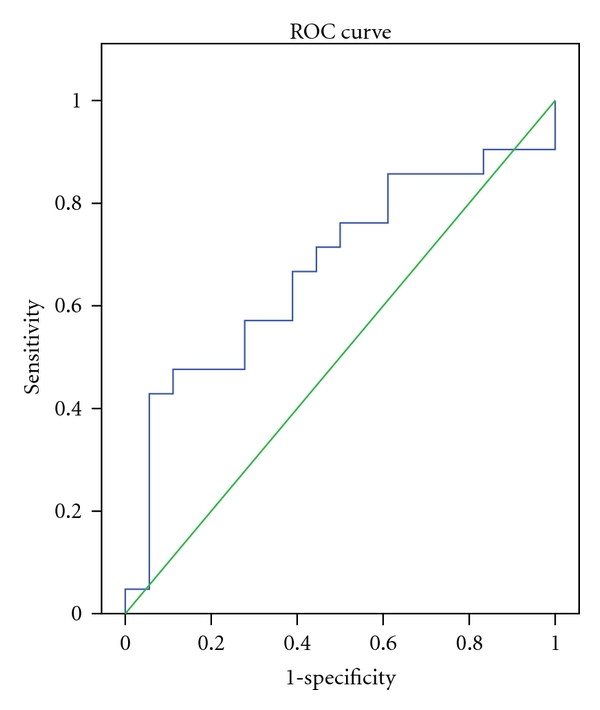
ROC curve of predictive value in significant liver fibrosis.

Sensitivity and specificity of Hepascore in diagnosis of significant fibrosis (F2, F3, and F4 from F0 and F1) in different cut-off points are shown in Figure 2. In cut-off point 0.34, sensitivity, specificity, PPV, and NPV were 67%, 56%, 64%, and 56%, respectively.

**Figure 3:**
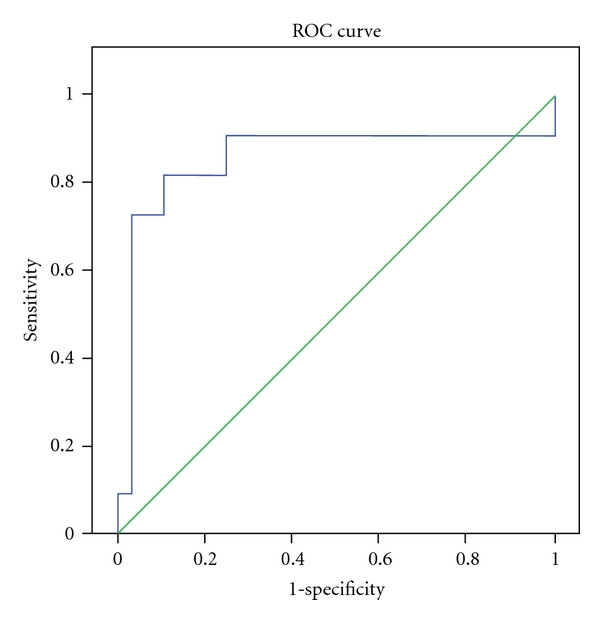
ROC curve of predictive value in diagnosis of severe fibrosis.

Sensitivity and specificity of Hepascore in diagnosis of severe fibrosis (F3 and F4 from F0, F1, and F2) in different cut-off points are demonstrated in Figure 3. In point 0.61, sensitivity, specificity, PPV, and NPV were 82%, 86%, 70%, and 92%, respectively.

**Figure 4:**
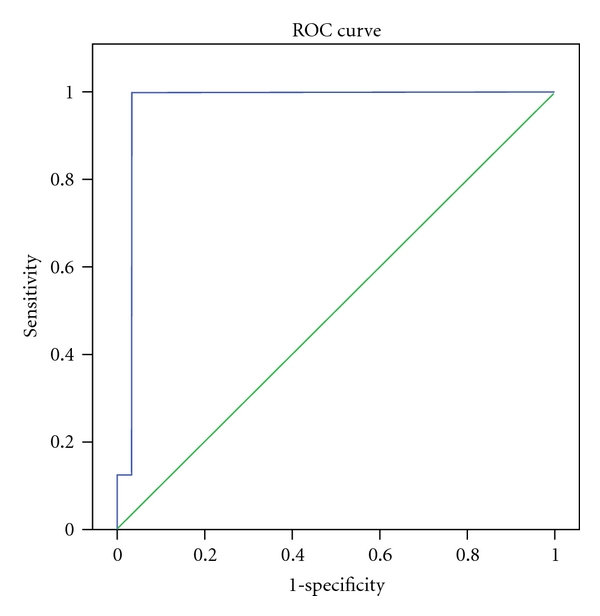
ROC curve of predictive value in diagnosis of cirrhosis.

Sensitivity and specificity of Hepascore in diagnosis of cirrhosis (F4 from F0, F1, F2, and F3) in different cut-off points are shown in Figure 4. In point 0.84, sensitivity, specificity, PPV, and NPV were 100%, 97%, 89%, and 100%, respectively.

## 4. Discussion

Prognosis of chronic liver diseases is strongly correlated with the degree of liver fibrosis. In chronic hepatitis C, besides having prognostic value, liver fibrosis is related to the therapeutic approach [15]. So far, no FDA-approved noninvasive method has been proposed for determination of liver fibrosis. A suggested and growing method is determination of Hepascore of the patients on the basis of blood markers of fibrosis. The current study demonstrated that Hepascore index has a reasonable sensitivity, specificity, NPV, and PPV. According to the results obtained, from among the cut-off points between 0 and 1, the most appropriate point for diagnosis of significant fibrosis from mild fibrosis and no fibrosis (F0 and F1) was 0.34. In other studies, cut-off points from 0.32 to 0.55 were obtained [1, 12, 14–16]. 

The authors who devised Hepascore [10] and also Adams et al. [12] methods suggested cut-off point 0.5. Becker et al. suggested point 0.55 as the most appropriate cut-off point [1], while Leroy et al. obtained cut-off point 0.32 [16] (Table 3).

Considering the above-mentioned points, the sensitivity of Hepascore in diagnosis of significant fibrosis was obtained to be point 67%. The value was reported to be from 54% to 82% [1, 12, 14]. Moreover, the specificity of the method was determined to be 56%, which was reported to be from 63% to 89% in previously performed studies [1, 10, 12, 16, 17]. The PPV and NPV of the test in diagnosis of significant fibrosis were determined to be 64% and 56%, respectively. In previous studies, the values were determined to be 59% to 89% and 64% to 80%, respectively [1, 14].

According to the results obtained, the most appropriate point for diagnosis of severe fibrosis from milder forms of fibrosis was determined to be 0.61, and the sensitivity and specificity were 82% and 86%, respectively. The appropriate cut-off points for this purpose were determined to be from 0.53 to 0.84 in previous studies.

We obtained the most appropriate cut-off point for diagnosis of cirrhosis for milder forms of fibrosis to be 0.84. At this cut-off point, sensitivity, specificity, PPV, and NPV were determined to be 100%, 97%, 89%, and 100%, respectively. Adams et al. [12] determined sensitivity and specificity of Hepascore in diagnosis of cirrhosis to be 71% and 84%, respectively. Guéchot et al. reported the sensitivity, specificity, PPV, and NPV of the test in diagnosis of cirrhosis to be 86%, 74%, 37%, and 97%, respectively. In spite of the differences among the values obtained in different studies, the high sensitivity and NPV were noteworthy in all the studies. The values obtained for these items were 100% in the current study. Therefore, using Hepascore, one can surely make decision on performance of screening for hepatocellular carcinoma, as well as carrying out endoscopy for evaluation of esophageal varicosis, both of which are currently rather high-cost and invasive procedures.

A factor that affects the above-mentioned elements is the difference in frequency distribution of severity of fibrosis in different studies. For instance, the prevalence of significant fibrosis in the current study was 53.8%, while the rate was reported to be 44% to 51% in other studies. Another cause for the differences is the errors in pathological interpretations and laboratory error. Nevertheless, since in clinical settings the aim of evaluation of these patients is detection of significant fibrosis to initiate the treatment, higher sensitivity of the test is of great importance.

As can be observed in Table 3, the findings of the current study are to a great extent consistent with other studies with regard to the relative weakness of Hepascore in diagnosis of lower stages of fibrosis and its high power in diagnosis of high stages of fibrosis. However, compared with previously performed studies, the cut-off point determined in the current study has a higher sensitivity and specificity for diagnosis of severe fibrosis and cirrhosis.

## 5. Conclusion

Hepascore is highly valuable in diagnosis of the severity of liver fibrosis and particularly cirrhosis (F4) and can be used as a primary screening method for diagnosis of the need for carrying out liver biopsy, which is a method with high costs and complications.

One of the limitations of the study was its small sample size, which was due to the economic constraints. With respect to the promising results obtained, similar study on a larger population can be performed.

## Figures and Tables

**Table 1:** Range of variation for independent variables.

Variable		Marker			
		HA ($\overset{̅}{X}\pm$ std error mean)	GGT ($\overset{̅}{X}\pm$ std error mean)	Bilirubin ($\overset{̅}{X}\pm$ std error mean)	*α* = *M* ($\overset{̅}{X}\pm$ std error mean)
Age group*	18–20 yr	34.7 ± 5.3	60 ± 10.9	32.9 ± 4.9	2.1 ± 0.15
	27–31 yr	23.6 ± 2.8	43 ± 4	15.3 ± 0.67	1.4 ± 0.12
	32–46 yr	21.8 ± 4.6	51.6 ± 6.2	15 ± 1.3	1.9 ± 0.2
	47–65 yr	96.1 ± 31.3	59.3 ± 7	16.5 ± 1.5	3.4 ± 0.2
*P* value for age**		0.003**	0.42	<0.001**	<0.001**

Sex***	Male	29 ± 3.34	56 ± 4.6	18.5 ± 1	2.1 ± 0.12
	Female	125.95 ± 46.33	41.6 ± 4.2	32.2 ± 9.6	3 ± 0.37
*P* value for sex**		<0.001**	0.19	0.004*	0.004*

Biopsy*	F0	31.5 ± 7	44.9 ± 7.1	31.5 ± 8.9	1.7 ± 0.1
	F1	16.25 ± 2.2	39.25 ± 5.4	18.8 ± 1.1	1.8 ± 0.19
	F2	26.16 ± 4.5	68.24 ± 8.8	13.27 ± 0.93	2 ± 0.24
	F3	30.6 ± 4.8	32.33 ± 3.1	11.9 ± 1.6	2.5 ± 0.52
	F4	121.97 ± 34.5	72.62 ± 10.2	27 ± 1.7	3.4 ± 0.14
*P* value for biopsy**		<0.001**	0.003**	0.002**	<0.001**

*Performed by one-way ANOVA.

**Significant at 0.05.

***Performed by independent  *t*-test.

**Table 2:** Frequency distribution for fibrosis severity according to the biopsy results.

Severity of fibrosis	No. of patients	%
F0	12	15
F1	25	31.25
F2	20	25
F3	7	8.75
F4	16	20

**Table 3:** Comparison of cut-off points, sensitivity, specificity, PPV, and NPV in different studies.

	Adams et al. [12]	Leroy et al. [16]	Halfon et al. [17]	Becker et al. [1]	Guechot et al. [14]	This study
Patients number	104	180	356	391	512	80
F0/F1/F2/F3/F4 (%)	16/27/34/7/16	8/41/22/14/14	4/55/26/11/4	16/34/15/16/19	7/45/18/15/15	15/31/25/8/20

For significant fibrosis						
Cut-off	0.5	0.5	0.32	0.55	0.5	0.34
Sensitivity	63%	54%	77%	82%	77%	67%
Specificity	89%	84%	63%	65%	70%	56%
Positive predictive value	/	78%	59%	70%	71%	64%
Negative predictive value	/	64%	80%	78%	77%	56%

For sever fibrosis						
Cut-off	/	0.84	0.53	0.8	0.6	0.61
Sensitivity	/	47%	78%	/	80%	82%
Specificity	/	90%	72%	77%	70%	86%
Positive predictive value	/	65%	32%	62%	54%	70%
Negative predictive value	/	81%	95%	/	89%	92%

For cirrhosis						
Cut-off	0.84	/	0.61	/	0.75	0.84
Sensitivity	71%	/	92%	/	86%	100
Specificity	89%	/	72%	/	74%	97%
Positive predictive value	/	/	11%	/	37%	89%
Negative predictive value		/	100%	/	97%	100
